# Mutant Isocitrate Dehydrogenase 1 Expression Enhances Response of Gliomas to the Histone Deacetylase Inhibitor Belinostat

**DOI:** 10.3390/tomography9030077

**Published:** 2023-05-04

**Authors:** Chi-Ming Chang, Karthik K. Ramesh, Vicki Huang, Saumya Gurbani, Lawrence R. Kleinberg, Brent D. Weinberg, Hyunsuk Shim, Hui-Kuo G. Shu

**Affiliations:** 1 Department of Radiation Oncology, Emory University, Atlanta, GA 30322, USA; 2 Department of Biomedical Engineering, Emory University and Georgia Institute of Technology, Atlanta, GA 30322, USA; 3 Department of Radiation Oncology, Johns Hopkin University, Baltimore, MD 21287, USA; 4 Department of Radiology and Imaging Sciences, Emory University, Atlanta, GA 30322, USA

**Keywords:** glioblastoma, HDAC, IDH, sMRI

## Abstract

Histone deacetylase inhibitors (HDACis) are drugs that target the epigenetic state of cells by modifying the compaction of chromatin through effects on histone acetylation. Gliomas often harbor a mutation of isocitrate dehydrogenase (IDH) 1 or 2 that leads to changes in their epigenetic state presenting a hypermethylator phenotype. We postulated that glioma cells with IDH mutation, due to the presence of epigenetic changes, will show increased sensitivity to HDACis. This hypothesis was tested by expressing mutant IDH1 with a point alteration—converting arginine 132 to histidine—within glioma cell lines that contain wild-type IDH1. Glioma cells engineered to express mutant IDH1 produced D-2-hydroxyglutarate as expected. When assessed for response to the pan-HDACi drug belinostat, mutant IDH1-expressing glioma cells were subjected to more potent inhibition of growth than the corresponding control cells. Increased sensitivity to belinostat correlated with the increased induction of apoptosis. Finally, a phase I trial assessing the addition of belinostat to standard-of-care therapy for newly diagnosed glioblastoma patients included one patient with a mutant IDH1 tumor. This mutant IDH1 tumor appeared to display greater sensitivity to the addition of belinostat than the other cases with wild-type IDH tumors based on both standard magnetic resonance imaging (MRI) and advanced spectroscopic MRI criteria. These data together suggest that IDH mutation status within gliomas may serve as a biomarker of response to HDACis.

## 1. Introduction

While the mutation of isocitrate dehydrogenase 1 (IDH1) at the arginine 132 residue (R132) was first identified via the whole-exome sequencing of a cohort of glioblastoma tumors [1], mutations of both IDH1 and IDH2 are now known to be present in a significant percentage of glial neoplasms, particularly lower-grade gliomas [2]. The point alteration in R132 in IDH1 or the comparable R172 residue in IDH2 changes the original enzyme activity that converts isocitrate to α-ketoglutarate (αKG) to one that now converts αKG to D-2-hydroxyglutarate (D-2HG) [3]. D-2HG has been termed an “oncometabolite” and can accumulate at very high levels (even into the mM range) in tumor cells harboring these IDH mutations [3,4]. These high levels of D-2HG have been shown to be detectable in IDH-mutant tumors through non-invasive means, such as MR spectroscopic techniques [5,6,7]. While high levels of D-2HG may have a range of activities, its main effects appear to be mediated by its ability to inhibit certain DNA- and histone-demethylating dioxygenases, which can alter the epigenetic state of cells [8,9,10]. Supporting this idea, the presence of IDH mutations has been associated with, and can, in fact, change, the epigenetic state of glioma cells, driving them towards a hypermethylator phenotype [11]. In addition to high levels of D-2HG, these epigenetic modulations in IDH-mutant gliomas result in widespread gene expression changes that can further alter the metabolic profile of these tumors, which are also potentially detectable using MR spectroscopy (for reviews, see [12,13]).

Histone deacetylases (HDACs) are a class of enzymes that act to remove acetyl groups from acetylated histones, resulting in epigenetic effects through the promotion of chromatin compaction [14]. The inhibition of HDAC activity has been shown to reverse this chromatin compaction and suppress tumor growth for a variety of malignant cell types, including gliomas [15,16,17]. Based on this, a class of drugs known as HDAC inhibitors (HDACis) have been developed that target these activities and have been shown to display significant antitumor effects in different malignancies [18,19]. To date, different HDACi drugs have been developed clinically and approved for use for indications including cutaneous and peripheral T-cell lymphomas and multiple myeloma [20]. Given the alterations in the epigenetic state of gliomas harboring mutant IDHs, it was conceivable that HDACi drugs may have increased activity in this tumor type.

Vorinostat, a first-generation pan-HDACi, has been assessed for the treatment of glioblastoma (GBM) patients, with limited success to date [21,22,23]. Belinostat, a later-generation pan-HDACi, displays higher potency than vorinostat and also penetrates the blood–brain barrier, making it a good candidate for the treatment of brain tumors [24,25]. We conducted a phase I trial evaluating the addition of belinostat to standard-of-care therapy for newly diagnosed GBM patients and found the maximum tolerated dose of belinostat when given in conjunction with radiation therapy (RT) and temozolomide (TMZ) chemotherapy [26]. In addition, the patterns of recurrence for belinostat-treated patients and the corresponding controls were carefully examined and resulted in the finding that the addition of this HDACi appears to enhance the central control of irradiated tumors [26]. Furthermore, the only GBM patient in this study with a tumor harboring the IDH1 R132H mutation (mtIDH1) appeared to display a highly enhanced response to the belinostat-containing treatment regimen [27]. Overall, these results suggest that the pan-HDACi belinostat may have activity in high-grade glial neoplasms, which warrants further testing of this drug, and that IDH mutational status may serve as a biomarker of response to HDAC inhibition.

To test the hypothesis that IDH-mutant glioma cells are more sensitive to HDAC inhibition, we generated glioma cell lines that expressed mtIDH1 and tested for sensitivity to HDAC inhibition, including with the pan-HDACi belinostat. Furthermore, we also tested for potential differences in the induction of apoptosis in the treated mtIDH1-expressing and the corresponding control cells. Finally, radiographic responses on our belinostat trial were recorded using standard magnetic resonance imaging (MRI) and spectroscopic MRI (sMRI), an advanced volumetric whole-brain MR spectroscopy technique, and assessed to determine differences in the response of a GBM harboring mtIDH1 versus GBMs with wild-type IDH (wtIDH) status.

## 2. Materials and Methods

### 2.1. Cell Lines and Culture Conditions

The normal human astrocyte (NHA) cell line used in this study was immortalized through the introduction of hTERT/large T antigen, as previously described [28], while the LN229 cell line is a long-established glioblastoma cell line that is available through the ATCC. These cells were cultured in high-glucose DMEM supplemented with 10% fetal bovine serum (FBS), sodium pyruvate (1 mM), and penicillin (100 units/mL)/streptomycin (100 μg/mL) (Sigma-Aldrich, St. Louis, MO, USA) unless otherwise indicated. The cDNA for wild-type IDH1 was changed via PCR-based site-directed mutagenesis using standard techniques to the IDH1 R132H mutant (mtIDH1)-altering codon 132 from 5′- …CGT…-3′ to 5′- …CAT…-3′ with sequence integrity confirmed using standard sequencing methods. The mtIDH1 gene-coding sequence was then inserted into the pBABE-Puro retroviral vector backbone to allow high-level expression under the control of the constitutive MLV promoter. The pBABE/mtIDH1 expression construct and pBABE alone without insertion were stably infected via standard techniques into NHA and LN229, followed by selection with puromycin to generate stable transfectants. Belinostat (Acrotech Biopharma, East Windsor, NJ, USA) stock solutions (10 mM) were made in DMSO and diluted to appropriate concentrations for experiments, as indicated.

### 2.2. Immunoblot Analyses

All immunoblots were carried out according to standard procedures. For the indicated assessments, the cells were lysed in standard buffers. Lysates with equal amounts of protein content were resolved using SDS–PAGE and electrotransferred to PVDF membrane (Bio-Rad, Hercules, CA, USA). Blots were probed with antibodies against IDH1 R132H mutant (MBL Life Sciences, Woburn, MA, USA), acetylated α-tubulin (Santa Cruz Biotechnology, Dallas, TX, USA), acetylated Histone-H4 (Santa Cruz), eIF5 (Santa Cruz), and GAPDH (Millipore Sigma, Burlington, MA, USA), according to the manufacturer’s recommendations. The appropriate horseradish peroxidase-conjugated secondary antibodies and reagents were used for chemiluminescent detection, according to manufacturers’ recommendations. After the application of the chemiluminescent substrate, the blots were exposed to standard X-ray film for appropriate lengths of time.

### 2.3. Liquid Chromatography–Tandem Mass Spectrometry (LC-MS/MS)

Conditioned media from the NHA and LN229 cell pairs (vector control and mtIDH1-expressing) were prepared by growing cells under standard conditions to 80% confluence on p100 culture plates. The cells were then washed with 1× PBS and cultured in 1.0 mL of fresh media containing 10% dialyzed fetal calf serum for 24 h. These conditioned media were then harvested and flash-frozen for storage prior to assessment using LC-MS/MS. The frozen samples were sent to the Emory Genetics Laboratory for processing and the D- and L- isoforms of 2HG were assessed in the conditioned media essentially as described by others [3,29].

### 2.4. Cell Growth and Apoptosis Assays

To assess for cell growth, direct cell counting, and MTT assays were used. Briefly, 50 K cells were seeded in each well of 24-well plates for the cell counting assay, while 50 K cells were seeded in each well of 6-well plates for the MTT assay. The indicated concentrations of belinostat in complete media were applied to cells 1 day after seeding, and fresh media containing appropriate levels of the drug were reapplied every 24 h for the duration of the experiment. The length of treatment/experiment was 48 h for the NHA pair and 72 h for the LN229 pair, after which the number of viable cells within the individual wells was directly determined via counting using a hemocytometer (cell count assay) or by incubating with MTT dye (Sigma) at 500 μg/mL in media without FBS for 1 h in a standard CO_2_ cell culture incubator (MTT assay). To measure the level of MTT staining, which is indicative of the number of living cells, MTT dye was removed, the cells were washed in the wells, and MTT dye was eluted from the cells still attached to the well using 0.5 mL of MTT elution buffer (4 mM HCl, 0.1% NP-40 in isopropanol). Optical density at 570 nM was measured to quantitate the level of MTT staining. For both the cell count and MTT assays, the results were normalized to cell counts/OD readings obtained with no drug, with these values arbitrarily set at 1.0.

Measurements of caspase activity levels and annexin V staining were used to assess the levels of apoptosis after belinostat treatment. For measurements of caspase activity, 4K cells were seeded in each well of 96-well plates, and belinostat at indicated concentrations was applied for 48 h starting 1 day after seeding. Cells were then harvested and assessed using the Caspase Glo 3/7 Assay System (Promega, Madison, WI, USA), according to the manufacturer’s recommendations. For the assessment of annexin V staining, 100 K cells were seeded in each well of 6-well plates, and belinostat at indicated concentrations was applied for 48 h starting 1 day after seeding. Cells were then harvested and assessed using the FITC Annexin V Apoptosis Detection Kit (BD Biosciences, San Jose, CA, USA) according to the manufacturer’s recommendation to measure the percent of annexin V positive (or apoptotic) cells after treatment. The results were normalized for these apoptosis assays to the level of caspase activity or % annexin V staining obtained with no drug with these values set at 1.0, as previously described.

### 2.5. Clinical Trial Information

Patients with newly diagnosed GBM were enrolled in this Institutional Review Board (IRB)-approved clinical trial at Emory and Johns Hopkins (ClinicalTrials.gov ID NCT02137759). The full results of this trial have been separately reported [26]. Patient tumor samples obtained from resection or biopsy were tested for IDH mutation. All patients were treated with standard-of-care therapy, which included radiation therapy and temozolomide chemotherapy along with intravenous belinostat (Acrotech Biopharma) for patients on the treatment arm as an investigational therapeutic, with full details of treatments described elsewhere [26]. Serial standard MRIs were obtained for patients pre-RT, 4 weeks post-RT, and at 2–3 month intervals unless clinical assessment dictated more frequent imaging. sMRI data were acquired for patients pre-RT and four weeks post-RT. sMRI data acquisition combined 3D echo-planar spectroscopic imaging with GRAPPA (generalized autocalibrating partial parallel acquisition) parallelization, and elliptical k-space encoding (TE/TR/FA = 17.6 ms/1551 ms/71°) on 3T MRI scanners with a 32-channel head coil array (Siemens Healthineers or Philips Healthcare). Metabolite maps were generated to create choline:N-acetyl aspartate (Cho:NAA) ratio maps normalized to values obtained from the contralateral normal-appearing white matter (NAWM); details of the processing pipeline have been described elsewhere [26]. This normalized Cho:NAA value is termed the Cho:NAA index (CNI).

## 3. Results

### 3.1. Expression of IDH1-R132H Mutant in Glioma Cell Lines

**Figure 1:**
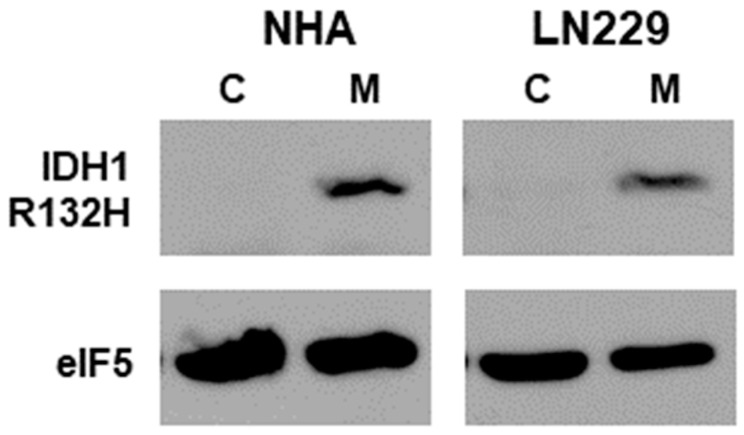
Immunoblots of lysates from the NHA and LN229 cell-line pairs (vector control (C) and mtIDH-expressing (M)) are shown. Upper blots were probed for the IDH1 R132H mutant, while the lower blots show detection of eIF5 as a loading control.

To assess the effect of mtIDH1 expression on glioma cells, the IDH1-R132H mutant was constitutively expressed in the NHA and long-established LN229 glioma cell lines, as described in Methods. These generated cell lines, along with corresponding vector-only control cells, were assessed for the expression of mtIDH1 through immunoblot analysis using an antibody that specifically binds to the R132H mutant protein. In each case, the expression of IDH1 R132H was only detected in the mutant IDH1-expressing cells and not in the corresponding vector-only controls (Figure 1). Next, the production of D-2HG by the mtIDH1-expressing cells was assessed using LC-MS/MS. For this assay, the NHA and LN229 cells expressing mtIDH1 and the corresponding vector-only controls were cultured, and the conditioned media from each line were generated as described in Methods. In each case, conditioned media were assessed for the presence of either the D- or L- isoforms of 2HG. In each case, elevated levels of D-2HG but not L-2HG were detected within media conditioned by mtIDH1-expressing NHA and LN229, while neither was elevated in media conditioned by the corresponding control NHA and LN229 (Table 1). These results show that our generated cells both express the R132H mutant form of IDH1 and produce high levels of D-2HG, the byproduct of mutant IDH.

### 3.2. Sensitivity of mtIDH1-Expressing and Control Glioma Cells to HDAC Inhibition

**Figure 2:**
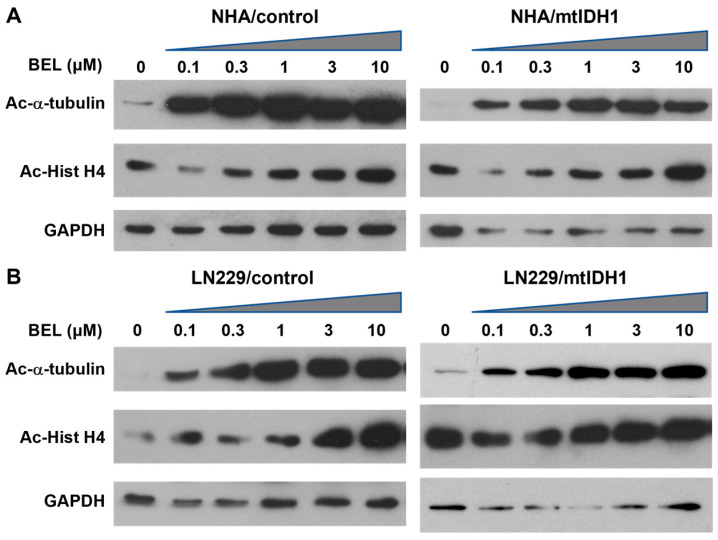
Immunoblots of lysates from the (**A**) NHA and (**B**) L229 cell-line pairs are shown. For each cell line, treatment was without the drug (0 μM) or increasing levels of belinostat (0.1, 0.3, 1.0, 3.0, and 10 μM) indicated above the lanes, with an ascending triangle signifying the progressively higher dose levels. Each group was probed for acetylated α-tubulin (Ac-α-tubulin) (upper blots) and acetylated Histone H4 (Ac-Hist H4) (middle blots). The lower blots show detection of GAPDH as a loading control.

To assess the mtIDH1-expressing and corresponding control cells for growth suppression via HDAC inhibition, each cell line was treated with increasing concentrations of the pan-HDACi belinostat. In each case, a dose-dependent increase in α-tubulin acetylation was found, based on immunoblot analyses, indicating that belinostat inhibited HDACs, as expected (Figure 2A,B, upper lanes). Increases in acetylated histone-H4 were also noted in response to belinostat, although the basal levels of acetylation observed in cells not treated with belinostat were found to be higher (Figure 2A,B, middle lanes). 

**Figure 3:**
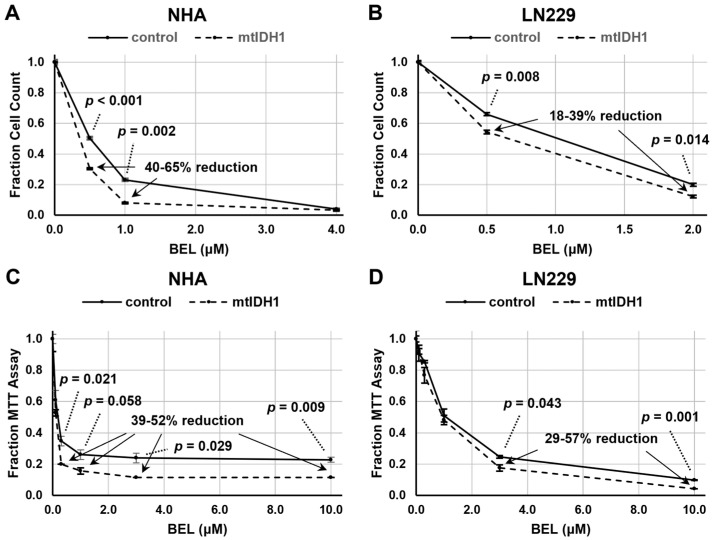
Assessments of cell growth after belinostat treatment were performed using either direct cell counting (**A**,**B**) or MTT assay (**C**,**D**). Results for control (solid lines) and mtIDH1 (dotted lines) cells were obtained in duplicate, with average values normalized as described in Methods. Error bars are ± one standard deviation for each data point. Significant *p*-values based on Student’s *t*-test and percent reduction ranges of the values of specific data points of mtIDH1-expressing versus control cells are shown on the graphs.

Next, these same cell lines were evaluated for growth suppression with increasing levels of belinostat. We initially assessed growth suppression based on the direct cell counts of NHA and LN229 isogenic cell-line pairs (vector-only controls and mtIDH1-expressing cells) and found a consistent decrease in the normalized cell count for the mtIDH1-expressing cells versus the control cells across a range of drug levels, indicating greater sensitivity to growth inhibition by belinostat (Figure 3A,B). We confirmed these results using an MTT assay, in which a consistent increase in growth suppression was also observed across a wide range of doses with the mtIDH1-expressing cells versus control cells (Figure 3C,D). With these assessments, the increase in the growth suppression of mtIDH1-expressing cells did appear to be less pronounced with LN229 than with NHA cells (Figure 3), consistent with the lower levels of D-2HG produced by the LN229/mtIDH1 cell line (Table 1). 

### 3.3. Glioma Cells Expressing mtIDH1 Display Increases Apoptosis with HDAC Inhibition

**Figure 4:**
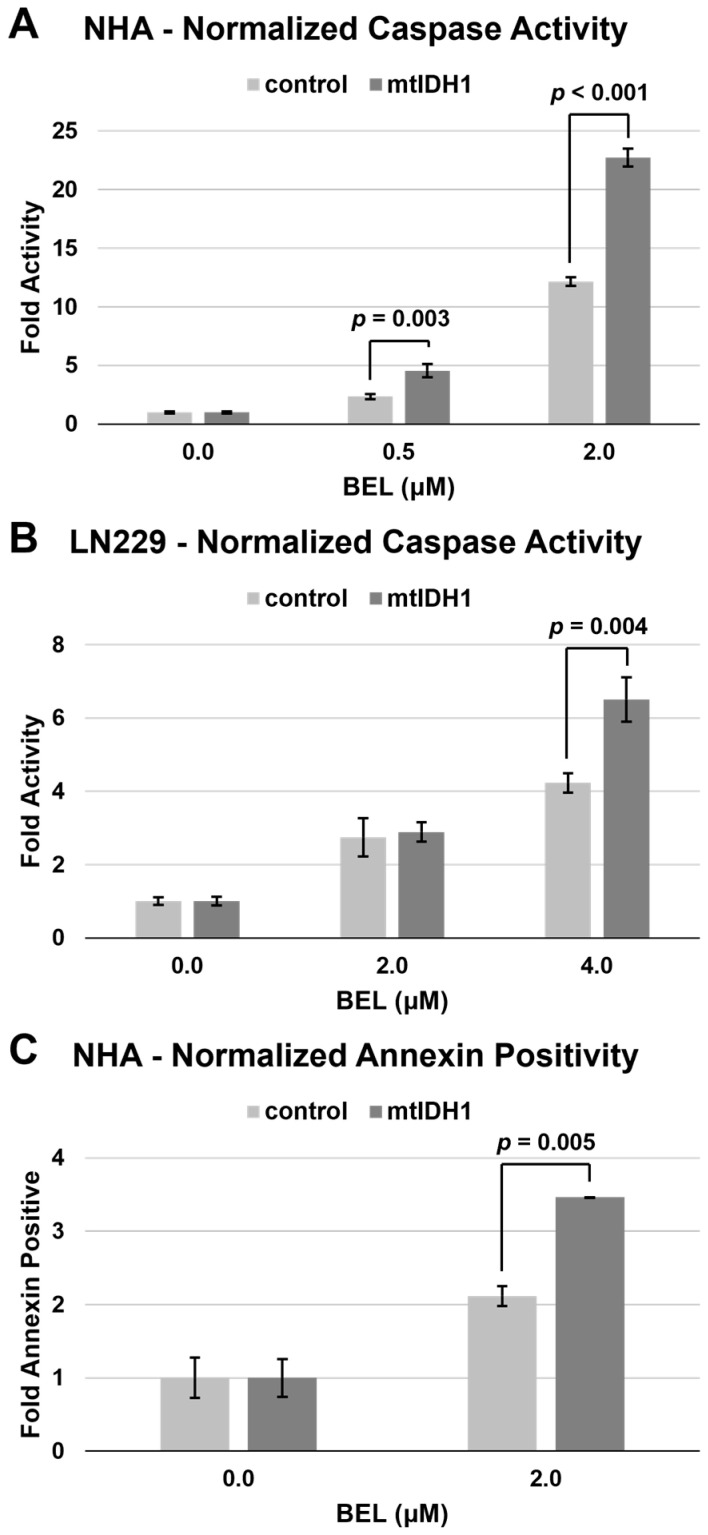
Assessment of apoptosis after belinostat treatment was performed by measuring either caspase activity (**A**,**B**) or fraction annexin V staining (**C**). Results for control (light bars) and mtIDH1 (dark bars) were obtained in duplicate, with average values normalized as described in Methods. Error bars are ± one standard deviation for each value. Significant *p*-values based on Student’s *t*-test for specific data points of mtIDH1-expressing versus control cells are shown on the graphs.

To determine the underlying means through which mutant IDH enhances the sensitivity of glioma cells to HDACis, we assessed for potential changes in the levels of apoptotic cell death in response to HDAC inhibition. We examined our isogenic NHA and LN229 cell-line pairs for the level of caspase activity after treatment with varying levels of belinostat. In each case, the mtIDH1-expressing cells showed a greater increase in the detected caspase levels, indicating a greater degree of apoptosis induction after treatment with belinostat in comparison with the corresponding vector control (Figure 4A,B). Next, we sought to assess the induction of apoptosis by another means, using an annexin V assay to confirm our results with the measurement of caspase activity. In this case, we opted to only assess the NHA vector control and mtIDH1 pair since this annexin V assay is a less sensitive assay, and NHA/mtIDH1 showed the highest production of D-2HG (Table 1). Using this assay, we found a significant increase in the induction of apoptosis after treatment with 2 μM of belinostat in the mtIDH1-expressing versus vector control NHAs (Figure 4C).

### 3.4. Mutant IDH1 GBM Shows Greater Response to a Belinostat-Containing Regimen than Corresponding Wild-Type IDH GBMs

**Figure 5:**
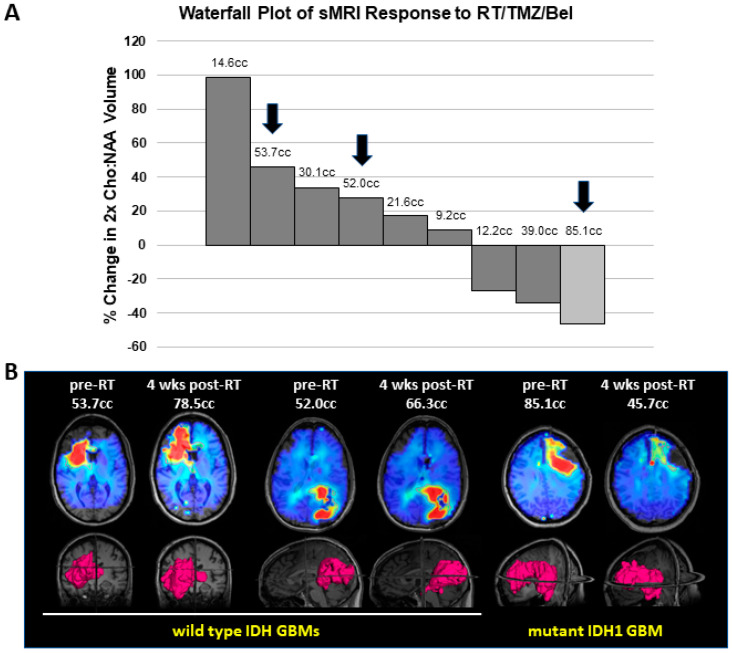
Assessment of sMRI response in GBM patients treated on our belinostat trial: (**A**) Waterfall plot for percent change in residual tumor between the pre-RT and 4-week post-RT sMRI scans, defined as volume of CNI values greater than 2. Nine patients had assessable sMRIs at both time points, comprising eight wild-type IDH GBMs (dark bars) and one mutant IDH1 GBM (light bar). Numbers above bars indicate the value of the pre-RT residual tumor volume. (**B**) The pre-RT and 4-week post-RT CNI maps for indicated slices on the 3D renderings for volumes of CNI values greater than 2 are shown for two wild-type IDH GBMs and one mutant IDH1 GBM. Arrows on the graph indicate the individual cases that are shown in this section.

**Figure 6:**
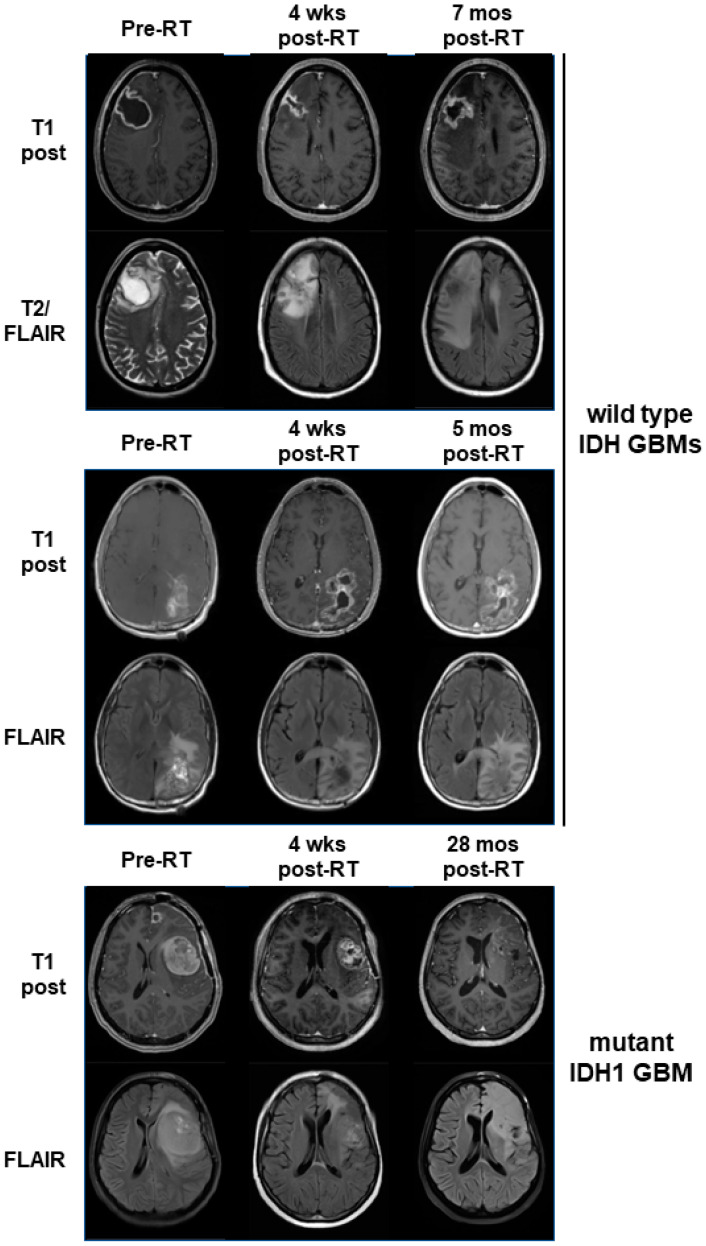
Serial standard MRIs (T1 post and FLAIR sequences) are shown for the same three patients illustrated in Figure 5B. Time points are as indicated, with the last time point being the scan where progression was officially called. One time point (upper panel, pre-RT) shows a T2 image as an alternative to a FLAIR image because a FLAIR sequence was not obtained at that time point.

To assess for response to belinostat in patients, we examined our cases in a phase I clinical trial where belinostat was added to standard-of-care RT/TMZ therapy for newly diagnosed GBMs (which has been separately reported) [26]. As part of this trial, all patients underwent sMRI, an advanced volumetric whole-brain MR spectroscopy technique whereby the remaining/residual tumor volume can be quantitatively determined. sMRI scans were performed both during pretreatment and approximately 4 weeks after the completion of RT/TMZ/belinostat. At the time of the trial, the WHO classification for GBMs did not exclude tumors harboring IDH mutations. Thus, 1 of the 13 patients on trial had a tumor with the IDH1 R132H mutation. This tumor could not be grossly resected due to its location in the L frontal lobe near the speech center. We compared the response of this tumor to the other tumors on trial for which we had usable quantitative sMRI data at the appropriate time points. We previously demonstrated that CNI (normalized Cho:NAA) values on sMRI show a direct pathological correlation with residual tumor cell density for GBM and may be elevated even in areas that do not display significant contrast enhancement on standard MRIs [30]. CNI values greater than 2 on sMRI were used as the threshold for significant residual disease. While our patient with a mtIDH1-expressing GBM had the highest volume of pretreatment residual tumor based on this definition, it also had the greatest sMRI response at 4 weeks post-RT when compared with the eight patients on trial for whom sMRI scans could be evaluated in both pre-RT and 4-week post-RT scans, as illustrated in our graph (Figure 5A). The CNI maps from pre-RT and 4 weeks post-RT are shown for two cases of wild-type IDH GBMs that had the highest volumes of pre-RT residual tumor (53.7 and 52.0 cc), as well as our mtIDH1-expressing case (85.1 cc pre-RT residual tumor), demonstrating the substantial differences in the observed sMRI response (Figure 5B). The greater sMRI response to a belinostat-containing regimen seen in our mutant IDH1 GBM versus the two residual tumor-matched wild-type IDH GBMs corresponds with these patients’ ultimate radiographic outcomes. When comparing their follow-up period in conventional MRIs, our wild-type IDH GBM cases showed clear tumor progression by 5–7 months post-RT (Figure 6, upper and middle panels) versus the initial regression of tumor followed by the overall control of the disease for greater than two years from the completion of RT in our mutant IDH1 GBM (Figure 6, lower panel).

## 4. Discussion

The results show that glioma cell lines that have been engineered to express mtIDH1 will produce the oncometabolite D-2HG and become more sensitive to growth inhibition by HDACis in a process that involves the enhancement of apoptotic response. Furthermore, we also found that a patient with a GBM harboring the IDH1 R132H mutation was exquisitely sensitive to a treatment regimen that included the pan-HDACi belinostat, especially when compared with other cases of GBM with wild-type IDH status. Thus, the presence of IDH mutation within glioma cells appears to render them more sensitive to HDAC inhibition. The presence of IDH mutation is generally considered to be a favorable prognostic factor for gliomas [31]. However, many such patients still progress and have poor clinical outcomes, especially for cases of higher-grade tumors. This supports the need for newer therapies, even for IDH-mutant gliomas, that can further improve patient outcomes. Thus, clinical testing exploring HDACis such as belinostat for IDH-mutant gliomas is warranted.

Sears et al. reported that panobinostat and vorinostat, both pan-HDAC inhibitors, were more effective in treating IDH-mutant patient-derived glioma cell lines than the corresponding IDH wild-type lines [32]. In addition, Dow et al. found that cancer cells (including the ones derived from gliomas) engineered to express mutant IDH1 showed increased sensitivity to vorinostat than the corresponding controls through the enhanced ability of this HDACi to suppress the homology-directed repair of DNA in IDH-mutant cells [33]. The reports from these other groups are consistent with our results indicating that IDH-mutant gliomas have greater sensitivity to HDAC inhibition. These findings together support the idea that IDH mutational status may serve as a biomarker of response to the HDAC inhibitor drug class.

While we have not elucidated the precise underlying mechanism for why IDH-mutant-expressing gliomas show an enhanced response to HDAC inhibition, we did find a more vigorous apoptotic response after treatment with an HDACi such as belinostat in the glioma cells engineered to express mtIDH1. This finding is consistent with the report by Dow et al., which showed impairment in homology-directed double-stranded DNA break repair for IDH1 mutant cells [33]. We would expect a greater apoptotic response if treatment with HDACis results in more DNA damage in mtIDH1-expressing glioma cells. Additionally, in this setting, we would expect enhanced DNA damage, particularly in conjunction with RT and TMZ chemotherapy, which may explain why our patient with a mutant IDH1 GBM responded so favorably to this therapy, especially if DNA repair machinery in the IDH-mutant tumor is impaired. While we can further explore whether there is enhanced DNA damage in our system, this is beyond the scope of our current report.

The assessment of serial sMRIs on our belinostat trial shows that the volume of the brain with CNI values greater than 2 reduced to the greatest extent with treatment in our case of a mutant IDH1 GBM. Besides the pathological correlation indicating that the elevated CNI values on sMRI reflect regions of the brain with significant GBM tumor cell infiltration [30], we have also shown that the sites of elevated CNI values appear to predict where GBMs will ultimately fail [34]. In addition, we conducted a three-site clinical trial using CNI on sMRI to help determine regions to target with RT dose escalation for GBM patients and found that both progression-free and overall survival were significantly increased compared with historical controls [35]. Finally, we have reported suggestive evidence that sMRI may actually be able to detect response to belinostat-containing therapy at an earlier time point than that observed on contrast-enhanced MRI [27]. All these studies provide support for the use of serial CNI maps obtained with sMRI scans as a quantitative means of tracking tumor response that may correlate with the ultimate clinical outcome.

As we previously indicated, D-2HG is an oncometabolite that is found at high levels in IDH-mutant gliomas [3]. In addition, the biological consequence of D-2HG production in gliomas is widespread epigenetic changes that can lead to altered tumor metabolism [4,8,10,11,36]. Therefore, the serial spectroscopic imaging of tumor metabolism through the assessment of specific metabolite levels may potentially be useful to track the response of IDH-mutant tumors. To date, we have primarily used CNI maps from our sMRI scans to identify tumors and track responses [30,35]. However, while changes in Cho and NAA levels may not be particularly specific for monitoring IDH-mutant tumors, alternative metabolites such as 2HG or other lower abundance ones may prove to be better, in particular, for tracking tumors harboring IDH mutation since they will have specific associated metabolic changes [37]. sMRI has the potential to provide whole-brain information on the levels of additional metabolites. Furthermore, additional sophisticated MR spectroscopic techniques such as 2D correlational spectroscopy (COSY) may allow better assessment of 2HG as well as other relevant less abundant metabolites. Therefore, further investigations in this area are warranted to fully realize the potential of spectroscopic imaging for IDH-mutant gliomas.

Our study does have some limitations. The use of established cell lines that contain other mutations irrelevant to IDH-mutant gliomas may confound the results. However, this possibility is mitigated to some extent by making comparisons of isogenic lines that should differ only by our introduced mtIDH1 expression vector. Furthermore, the NHA cell line that we used should contain relatively few additional mutations since it was originally generated by expressing hTERT and large T antigen in normal astrocytes. Additional assessment using patient-derived glioma cells with intrinsic IDH1 mutation would add more strength to our findings and is being pursued, but it is beyond the scope of this current report. Finally, we acknowledge that our demonstration that a mtIDH1 GBM will respond robustly to a belinostat-containing treatment regimen represents only one case, so statistical significance cannot be ascertained. However, this patient example, along with our preclinical evidence, lends credence to further clinical testing of this approach.

In summary, the expression of mutant IDH1 in glioma cells increases their sensitivity to treatment with an HDACi such as belinostat; this is further supported by the finding that a patient with a GBM harboring an IDH1 mutation also showed a much greater response to a belinostat-containing treatment regimen than other wild-type IDH GBMs in our clinical trial. Overall, these results are highly suggestive that glial tumors with IDH mutation have enhanced sensitivity to a treatment regimen that includes an HDACi such as belinostat. These findings support future clinical testing of HDACis such as belinostat for IDH-mutant gliomas to potentially improve clinical outcomes.

## Figures and Tables

**Table 1:** Detection of D- and L-2HG using LC-MS/MS.

Sample ^1^	D-2HG (μM)	L-2HG (μM)
NHA		
vector control	0.07	0.10
mtIDH1	18.50	0.06
LN229		
vector control	0.06	0.03
mtIDH1	5.85	0.05
Spiked with specific levels		
50 μM (D- or L-2HG)	58.00	53.30
100 μM (D- or L-2HG)	115.00	120.00

^1^ LC-MS/MS, liquid chromatography–tandem mass spectrometry; NHA, immortalized normal human astrocytes; mtIDH1, mutant isocitrate dehydrogenase.

## Data Availability

The data presented in this study are available on request from the corresponding author.
